# A Study on Changes in Serum HMGB1/RAGE Pro-Inflammatory Axis and Related Cytokines Levels in Patients with Acute Brucellosis in Qinghai Province, China

**DOI:** 10.3390/tropicalmed11100289

**Published:** 2026-10-13

**Authors:** Yangyang Chen, Jianling Wang, Qiang Li, Yanmei Zhao, Hongmei Xue, Jiquan Li, Cunzhe Zhao, Li Ma, Shan Li, Shuai Tang, Xun Chen, Zhijun Zhao

**Affiliations:** Qinghai Institute for Endemic Disease Prevention and Control, Xining 810021, China

**Keywords:** brucellosis, high mobility group box 1, receptor of advanced glycation end products, inflammation

## Abstract

**Objective:** The serum levels of high mobility group box 1 (HMGB1), the receptor for advanced glycation end products (RAGE), endogenous secretory RAGE (esRAGE), S100 calcium-binding protein A12 (S100A12), and advanced glycation end products (AGEs) were measured in patients with acute brucellosis and control subjects to elucidate the association between brucellosis and the HMGB1/RAGE pro-inflammatory axis, ultimately providing more scientific evidence for the prevention and control of the disease. **Methods:** A total of 112 patients with acute brucellosis were enrolled as the case group, and 119 healthy individuals were enrolled as the control group. Serum levels of HMGB1, RAGE, esRAGE, S100A12, and AGEs were determined by enzyme-linked immunosorbent assay (ELISA). The differences in serum levels of each indicator between groups were compared, and Pearson correlation analysis was employed to explore the correlations among HMGB1, RAGE, esRAGE, S100A12, and AGEs in the serum of brucellosis patients. **Results:** The serum level of HMGB1 in the case group was (9.188 ± 3.249) ng/mL, and that in the control group was (7.687 ± 2.385) ng/mL. A statistically significant difference in serum HMGB1 level was observed between the two groups (*t* = 4.019, *p* < 0.05). The serum level of RAGE in the case group was (3.847 ± 0.940) ng/mL, and those in the control group were (3.480 ± 0.930) ng/mL. A statistically significant difference in serum RAGE level was found between the two groups (*t* = 2.986, *p* < 0.05). The serum level of AGEs in the case group was (325.5 ± 144.2) μg/mL, and those in the control group were (356.6 ± 84.5) μg/mL. A statistically significant difference in serum AGE level was detected between the two groups (*t* = −2.009, *p* < 0.05). No statistically significant differences in serum levels of esRAGE and S100A12 were identified between the two groups. **Conclusions:** Serum levels of HMGB1, RAGE, and AGEs were found to be abnormally altered in patients with acute brucellosis. These changes suggest that HMGB1, RAGE, and AGEs may play a role in the host’s early immune response to *Brucella*. However, as this study only measured the circulating concentrations of these cytokines, further mechanistic studies are required to verify whether the observed alterations are involved in the signal transduction pathways of the *Brucella*-induced inflammatory response in the host.

## 1. Introduction

Brucellosis is a zoonotic infectious disease caused by the genus *Brucella*, which is primarily transmitted through direct contact with infected animals, consumption of undercooked meat, or ingestion of unpasteurized dairy products [1]. Globally, there are approximately 500,000 new cases of brucellosis each year, and the actual incidence rate is significantly higher than the reported number of cases [2]. In China, the number of brucellosis cases has increased rapidly since the mid-1990s, making it one of the fastest-growing infectious diseases [3]. According to the latest report from the Chinese Center for Disease Control and Prevention (China CDC), a total of 69,767 human brucellosis cases were reported nationwide in 2021, representing a 47.7% increase compared to 2020 [4]. The clinical manifestations of brucellosis are complex and diverse; among them, acute-phase cases are mainly characterized by fever, excessive sweating, fatigue, arthralgia, and other symptoms [5]. *Brucella* is a Gram-negative, facultative intracellular aerobic bacterium that primarily parasitizes macrophages in the human body. It spreads through self-proliferation, invades the lymphatic system, and subsequently invades various organs and systems throughout the body, triggering extensive inflammatory responses in the organism [6].

As a widely distributed structural protein in the human body, HMGB1 comprises approximately 215 amino acids and is ubiquitously expressed in diverse cell types. When intracellular HMGB1 was secreted extracellularly, it could act as a pro-inflammatory factor to participate in the initiation of inflammation [7]. The functions of HMGB1 in inflammatory responses are mainly manifested as follows [8]: ① promoting immune cells to migrate to the site of injury; ② enhancing the recognition of antigens such as bacteria by innate immune cells; ③ activating innate immune cells to produce more pro-inflammatory factors and exacerbate the inflammatory response; ④ inhibiting the programmed cell death of neutrophils, leading to the accumulation of pro-inflammatory factors. Extracellularly, HMGB1 mainly binds to RAGE and Toll-like receptors (TLRs). Among these, HMGB1 has high affinity for RAGE, and their interaction can induce various cells to generate a series of complex biological effects [9,10].

RAGE is a transmembrane receptor belonging to the immunoglobulin superfamily, and it was also the first receptor identified to bind to HMGB1 [11]. In most disease states, the serum level of RAGE increases. Through interaction with ligands, it participates in various physiological processes, including immune response, oxidative stress, inflammation promotion, and angiogenesis [12]. RAGE in the human body can be expressed in various types of cells, such as vascular epithelial cells, fibroblasts, and different immune cells. These RAGE molecules distributed on the cell surface can bind to a variety of ligand molecules, thereby activating various intracellular signal transduction pathways, regulating a variety of physiological and biochemical reactions, and exerting specific functions [13].

At present, research on brucellosis mainly focuses on epidemiological investigations and etiological studies [14]. To this end, this study aims to measure the serum levels of the HMGB1/RAGE pro-inflammatory axis and related cytokines in patients with acute brucellosis, to observe changes in the patients’ inflammatory responses, and to elucidate the correlation between brucellosis and the aforementioned cytokines, thereby providing a stronger scientific basis for the prevention and treatment of the disease.

## 2. Materials and Methods

### 2.1. Study Subjects

In accordance with “Diagnostic Criteria for Brucellosis (WS 269-2019)” and the principle of informed consent, acute brucellosis patients who visited the brucellosis clinic of Qinghai Institute for Endemic Disease Prevention and Control from July to August 2022 were enrolled as the case group. Meanwhile, healthy individuals were matched as the control group, with a total of 231 subjects surveyed. Among them:

The case group consisted of 112 patients (71 males and 41 females), aged (45.86 ± 10.485) years (range: 23–62 years), who were diagnosed with acute brucellosis through clinical examinations and laboratory tests.

The control group included 119 subjects (70 males and 49 females), aged (43.63 ± 10.802) years (range: 26–68 years), with certain similarities to the case group in terms of living standards, living habits, and other aspects.

This study was supervised by the medical ethics committee of Qinghai Institute for Endemic Disease Prevention and Control. Informed consent forms were signed, and the obligation of information disclosure was fulfilled. The research was conducted only with the consent of the subjects.

The case group consisted of patients with acute brucellosis. The inclusion criteria were: history of epidemiological exposure, typical clinical manifestations, positive results in both the Rose Bengal plate test (RBPT) and the serum agglutination test (SAT), and disease duration within 3 months. The exclusion criteria were: receiving prior drug treatment, having other bacterial or viral febrile diseases, a prior history of smoking, and incomplete baseline data.

Based on the SAT antibody titers, patients in the case group were further divided into three subgroups for subgroup analysis: a low-titer group (SAT 1:100), a medium-titer group (SAT 1:200), and a high-titer group (SAT 1:400).

The control group comprised healthy individuals. The inclusion criteria were no epidemiological exposure history, no clinical symptoms, and negative results in both RBPT and SAT. The exclusion criteria were chronic diseases, immune-mediated diseases, other severe medical or surgical diseases, a prior history of smoking, and incomplete baseline data.

### 2.2. Experimental Methods

#### 2.2.1. General Demographic Data

In accordance with the formulated survey protocol, staff from the brucellosis department of Qinghai Institute for Endemic Disease Prevention and Control conducted one-on-one interviews with subjects in both the case group and the control group at the department’s outpatient clinic [15]. General demographic data of all respondents, including age, gender, ethnicity, occupation, and place of residence, were recorded in strict adherence to the format and requirements of the questionnaire.

#### 2.2.2. Detection of Serum Levels of Cytokines in the Target Population

Fasting elbow venous blood was collected from the target population (fasting blood was collected to prevent serum dilution or turbidity caused by lipemia, which might affect the laboratory results). Blood samples from the target population were collected in pyrogen-free and endotoxin-free tubes. The blood samples were allowed to stand at room temperature for 30 min, then centrifuged at 4000 rpm for 5 min to rapidly separate serum from red blood cells. After centrifugation, the supernatant was collected, aliquoted into cryovials according to single-use volumes with proper labeling, and stored at −80 °C for subsequent use. Prior to detection, the samples were thawed at room temperature to ensure complete and uniform thawing. ELISA was used to determine the concentrations of various cytokines (kits purchased from Beijing Airuahua Technology Co., Ltd., Beijing, China). According to the manufacturer’s protocols (the detection ranges were 1.0–200 nmol/L, 1.0–80 μg/L, 0.1–8 μg/L, respectively, and intra-and inter-assay CVs were less than 9% and 15%). Specifically, the RAGE ELISA kit detects total soluble RAGE (sRAGE) in serum, which comprises both the endogenous secretory isoform (esRAGE) and the soluble receptor generated through proteolytic cleavage of cell-surface RAGE (cleaved RAGE). In contrast, the esRAGE kit is designed to exclusively detect the endogenous secretory isoform.

### 2.3. Statistical Analysis

Laboratory data were collated and organized using Excel 2003 (Microsoft Corporation, Redmond, WA, USA), and statistical analyses were performed with SPSS 17.0 (SPSS Inc., Chicago, IL, USA). Normality tests were conducted for quantitative data:

If the data followed a normal distribution, statistical descriptions were presented as $\bar{x}$ ± s (mean ± standard deviation).

If the data showed a skewed distribution, the median and interquartile range were used for statistical descriptions.

The independent-samples *t*-test was used for comparison between two groups. When the data did not meet the assumptions of the *t*-test, the nonparametric test (Wilcoxon rank-sum test) was applied instead. One-way ANOVA was used for comparisons among multiple groups, and the LSD method was used for post-hoc pairwise comparisons when significant differences were observed among groups. Categorical variables were described by frequencies and percentages, and comparisons of count data were performed using the chi-square test.

Pearson correlation analysis was used to explore the relationships among serum cytokines (HMGB1, RAGE, esRAGE, S100A12, AGEs, etc.) in brucellosis patients. Prior to analysis, normality tests were conducted for all variables, and graphical methods were employed to assess linearity and the assumption of no influential outliers. Since the data satisfied these assumptions, Pearson correlation analysis was selected; otherwise, Spearman’s rank correlation analysis was used as an alternative. The correlation strength was defined as follows:

Positive correlation when r > 0, negative correlation when r < 0;

Weak correlation if 0 ≤ |r| < 0.3;

Moderate correlation if 0.3 ≤ |r| < 0.6;

Strong correlation if 0.6 ≤ |r| < 1.

A significance level of α = 0.05 was set, and a *p*-value < 0.05 was considered statistically significant.

Given the extensive number of comparisons and correlation analyses conducted in this study (including subgroup analyses across antibody titer levels), we accounted for the potential inflation of the Type I error rate due to multiple testing. To address this in our sensitivity analysis, we applied the Benjamini–Hochberg (BH) procedure to control the False Discovery Rate (FDR).

## 3. Results

A total of 231 subjects were surveyed. There was no statistically significant difference in age between the case group and the control group (*t* = 1.588, *p* = 0.114). Among the 112 patients in the case group, there were 71 males (64.0%) and 41 females (36.0%), with a male-to-female ratio of approximately 1.73:1. There was no statistically significant difference in gender between the two groups (χ^2^ = 0.507, *p* = 0.477). There was no statistically significant difference in ethnicity distribution between the two groups (χ^2^ = 8.772, *p* = 0.187). A chi-square test was performed on the occupation distribution of the two groups, and the difference was statistically significant (χ^2^ = 15.100, *p* = 0.019). See Table 1.

The clinical manifestations of brucellosis patients included fever, chills, excessive sweating, fatigue, migratory arthralgia (involving systemic joints such as the knee joint, hip joint, and shoulder joint), lumbago, myalgia, neuralgia, and testicular swelling and pain. See Table 2.

All RBPT results of the case group were positive, and the SAT antibody titers were all ≥ 1:100. Both RBPT and SAT results of the control group were negative. See Table 3.

**Figure 1:**
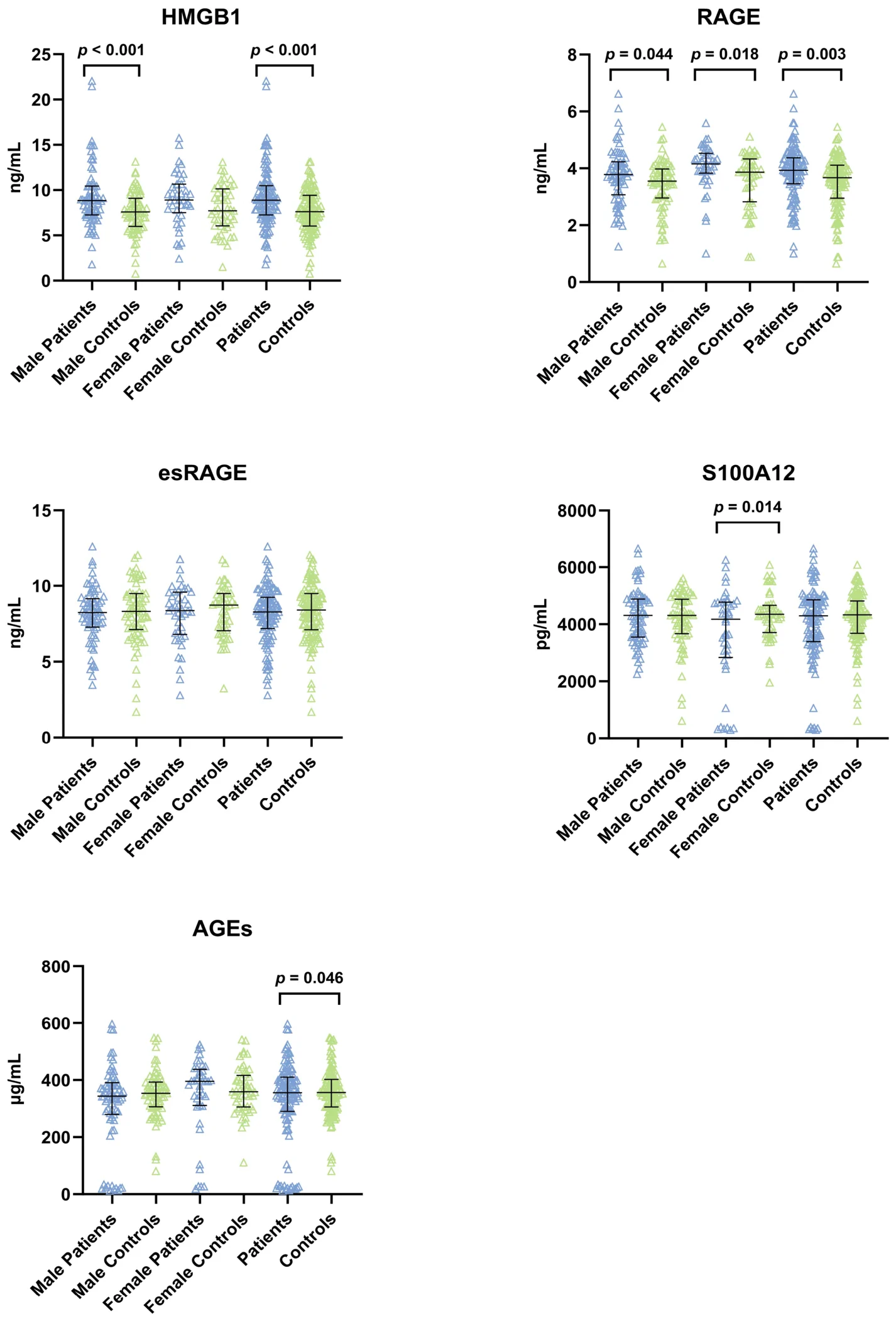
Distribution of serum levels of HMGB1, RAGE, esRAGE, S100A12, and AGEs between the case group and the control group.

The serum levels of HMGB1, RAGE, and AGEs in the case group were (9.188 ± 3.249) ng/mL, (3.847 ± 0.940) ng/mL, and (325.5 ± 144.2) μg/mL, respectively, with a statistically significant difference in serum HMGB1, RAGE, and AGEs between the two groups (all *p* < 0.05). The serum levels of esRAGE and S100A12 in the case group were (8.085 ± 1.830) ng/mL and (4052 ± 1311) pg/mL, respectively. There were no statistically significant difference in serum levels of esRAGE and S100A12 between two groups (all *p* > 0.05). See Table 4 and Figure 1.

**Figure 2:**
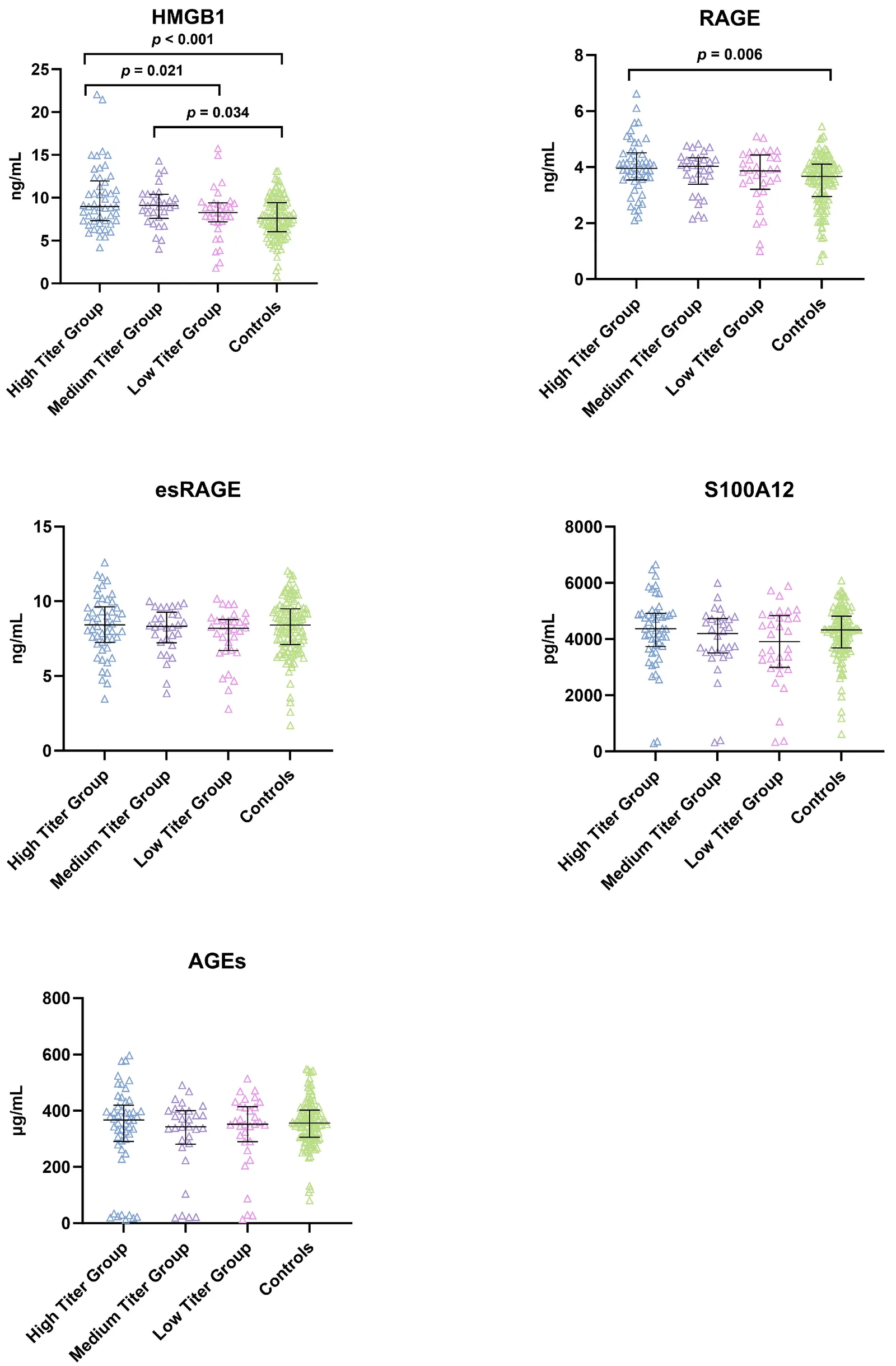
Distribution of serum levels of HMGB1, RAGE, esRAGE, S100A12, and AGEs between the case group with different antibody titers and the control group.

ANOVA was performed on the serum level of HMGB1 in different titer subgroups, with a statistical difference (F = 7.684, *p* < 0.05). ANOVA was performed on the serum level of RAGE in different titer subgroups, with a statistical difference (F = 3.957, *p* < 0.05). ANOVA was performed on serum levels of esRAGE, S100A12, and AGEs across different titer subgroups, with no statistically significant difference observed. See Table 5 and Figure 2.

Correlation analysis revealed that serum HMGB1 levels in patients with brucellosis were positively correlated with RAGE (r = 0.447, *p* < 0.05) and esRAGE (r = 0.288, *p* < 0.05), but negatively correlated with AGEs (r = −0.216, *p* < 0.05); Serum RAGE levels were positively correlated with both esRAGE (r = 0.602, *p* < 0.05) and AGEs (r = 0.358, *p* < 0.05); Serum esRAGE levels showed a positive correlation with AGEs (r = 0.587, *p* < 0.05). In the high antibody titer subgroup of patients with brucellosis, serum HMGB1 levels were negatively correlated with AGEs (r = −0.363, *p* < 0.05); serum RAGE levels were positively correlated with both esRAGE (r = 0.512, *p* < 0.05) and AGEs (r = 0.339, *p* < 0.05); serum esRAGE levels showed a positive correlation with AGEs (r = 0.556, *p* < 0.05). In the moderate antibody titer subgroup of patients with brucellosis, serum HMGB1 levels were positively correlated with RAGE (r = 0.495, *p* < 0.05) and esRAGE (r = 0.408, *p* < 0.05); serum RAGE levels were positively correlated with both esRAGE (r = 0.540, *p* < 0.05) and AGEs (r = 0.391, *p* < 0.05); serum esRAGE levels showed a positive correlation with AGEs (r = 0.616, *p* < 0.05). In the low antibody titer subgroup of patients with brucellosis, serum HMGB1 levels were positively correlated with RAGE (r = 0.666, *p* < 0.05), esRAGE (r = 0.566, *p* < 0.05), and S100A12 (r = 0.412, *p* < 0.05); serum RAGE levels were positively correlated with esRAGE (r = 0.763, *p* < 0.05); serum esRAGE levels showed a positive correlation with AGEs (r = 0.648, *p* < 0.05). See Table 6, Table 7, Table 8 and Table 9.

The current results indicate that: Compared with the healthy control group, patients with acute brucellosis exhibited significantly higher serum levels of HMGB1 and RAGE, but lower levels of AGEs. No significant differences were observed in the levels of esRAGE and S100A12. Both HMGB1 and RAGE are cytokines involved in the host’s inflammatory response; their elevated levels suggest a more pronounced inflammatory state during acute *Brucella* infection. The decreased level of AGEs may be associated with biological alterations related to the acute inflammatory response. These findings are merely preliminary observational results. Future mechanistic validation experiments, cohort studies, and health economic analyses are required to determine whether these biomarkers can be incorporated into routine clinical practice.

## 4. Discussion

Brucellosis is a zoonotic infectious disease caused by Gram-negative *Brucella* invading the body through multiple routes, with the first report in China in 1905 [16]. It is distributed worldwide, with a peak incidence from March to May. The disease is mainly endemic in regions with concentrated cattle and sheep breeding but relatively backward economic and technological development, such as the Middle East, Latin America, Asia, Africa, and the Mediterranean basin. The affected population is predominantly young and middle-aged adults, and the prevalence rate is higher in males than in females [17]. As one of China’s four major pastoral areas, Qinghai province has a long history of brucellosis prevalence and is a high-incidence province for human and animal brucellosis in China. From 2005 to 2010, the incidence rate of brucellosis in the province ranged from 0 to 0.05 per 100,000 population; from 2011 to 2018, it ranged from 0.27 to 2.06 per 100,000 population [18]. From 2019 to 2020, a total of 11,707 people were monitored at provincial brucellosis surveillance sites, with 236 positive results for RBPT, 150 positive results for SAT, and 139 confirmed cases, resulting in a prevalence rate of 1.19% [19].

After *Brucella* invades the body, it can invade multiple tissues and organs, thereby triggering relevant immune responses. Firstly, macrophages and neutrophils cannot effectively and completely clear *Brucella* from the body. Subsequently, the remaining *Brucella* continues to stimulate the activation of CD4+ T cells, B lymphocytes, and other cells, inducing adaptive immune responses. These activated cells secrete antibodies, complement, and cytokines of T helper cell 1 (Th1) and T helper cell 2 (Th2) types to exert bactericidal effects [20]. As intracellular bacteria, *Brucella* regulates and escapes host immunity through intracellular parasitism and replication, hindering its clearance by the host. Therefore, the host’s clearance of *Brucella* mainly relies on cellular immunity.

Previous studies have demonstrated that the HMGB1/RAGE pro-inflammatory axis and related cytokines are involved in the signal transduction pathways of various inflammatory responses. The binding of HMGB1 to RAGE can induce one of the key pathways of intracellular signal transduction, namely the mitogen-activated protein kinase (MAPK) pathway. Through this pathway, the HMGB1/RAGE signaling axis promotes the production of cytokines and chemokines, thereby participating in the differentiation and migration of immune cells as well as the expression of cell surface receptors, ultimately leading to related tissue damage and inflammation [21].

HMGB1 is a dual-function molecule in the human body. It serves as both a structural protein essential for maintaining vital activities in the cell nucleus and a key signaling molecule that mediates inflammatory and immune responses in the extracellular environment. When cells undergo necrosis due to physical injury, ischemia, infection, or other causes, the integrity of the cell membrane is disrupted, and HMGB1 originally located in the nucleus is passively released into the extracellular space. Various immune cells, such as activated macrophages, monocytes, dendritic cells, etc., actively secrete HMGB1 into the extracellular environment upon stimulation by pathogen components (e.g., lipopolysaccharide, LPS) or other stressors [22]. In the internal environment, HMGB1 binds to RAGE and activates the p38 MAPK and extracellular regulated protein kinase 1/2 (ERK1/2) signaling pathways, regulating the secretion of more RAGE, TNF, vascular endothelial growth factor (VEGF), and thymic stromal lymphopoietin (TSLP). Upon binding to TLRs, HMGB1 activates myeloid differentiation factor 88 (MyD88) and the related nuclear factor kappa-B (NF-κB) pathway, which in turn promotes the massive release of various inflammatory mediators including interleukin-1 (IL-1), IL-6 and TNF, ultimately leading to the occurrence of inflammatory responses [23]. As facultative intracellular Gram-negative bacteria, *Brucella* extensively colonizes the mononuclear phagocyte system in the liver, spleen, and bone marrow, continuously releasing antigenic substances such as lipopolysaccharides, outer membrane proteins, and bacterial nucleic acids. This intense and continuous antigenic stimulation polarizes mononuclear macrophages toward an M1 pro-inflammatory phenotype. As core cells actively secreting HMGB1, M1 macrophages release nuclear HMGB1 into the extracellular space via non-classical vesicular pathways, directly elevating peripheral blood HMGB1 levels [24]. During the acute phase of brucellosis, patients with medium to high antibody titers harbor massive *Brucella* proliferation, which compromises the integrity of macrophages, hepatocytes, and endothelial cells. This induces apoptosis and necrosis in infected cells, leading to karyorrhexis and a massive release of nuclear-stored HMGB1 into the bloodstream. Furthermore, macrophage-derived HMGB1 can bind to TLR2, TLR4, and RAGE receptors, thereby activating the NF-κB and MAPK signaling pathways. This activation triggers the massive secretion of pro-inflammatory cytokines, including TNF-α, IL-1β, and IFN-γ. These inflammatory cytokines, in turn, further stimulate macrophages to synthesize and release more HMGB1, forming a cascade amplification effect. Consequently, serum HMGB1 levels in the high- and medium-titer groups of acute-phase brucellosis patients are higher than those in the control group. Conversely, patients with low titers are typically in the early stage of infection, before the full expansion of bacteremia. Due to the low pathogen load, limited release of pathogen-derived stimuli, and restricted macrophage activation, no significant difference in serum HMGB1 levels is observed between these patients and the control group. These findings suggest that HMGB1 could serve as a biomarker reflecting the degree of inflammatory activation and tissue damage during acute *Brucella* infection.

As a transmembrane receptor, RAGE can interact with various ligands, such as S100/calgranulins, AGEs, HMGB1, and β-amyloid protein (Aβ) [25]. RAGE is capable of recognizing unique molecular patterns produced during viral and bacterial invasion, namely pathogen-associated molecular patterns (PAMPs) and damage-associated molecular patterns (DAMPs). The recognition of these two molecular patterns by RAGE facilitates the activation of innate immune responses; in addition, this receptor can promote and regulate adaptive immune responses through DCs or T cells [11]. RAGE plays a central role in the signaling pathways that regulate cell apoptosis and the clearance of apoptotic cells, which is crucial for maintaining the stability of the immune system [26]. It can also regulate immune functions through inflammasome signaling pathways [27]. After phagocytosing and processing apoptotic cells, DCs present antigens to CD8+ T cells via the major histocompatibility complex class I (MHC I) pathway, thereby inducing immune tolerance [28]. The NOD-like receptor protein 3 (NLRP3) inflammasome in DCs can activate cysteinyl aspartate-specific proteinase-1 (Caspase-1) to promote HMGB1 release [29]. HMGB1 can reverse apoptosis-induced immune tolerance through the interaction between its A box and RAGE. On the other hand, within macrophages, RAGE can activate the absent in melanoma 2 (AIM2) inflammasome, leading to increased expression levels of the pro-inflammatory factors HMGB1, interleukin-1β (IL-1β), and interleukin-18 (IL-18) [30]. *Brucella* LPS directly regulated the expression of the RAGE gene through the TLR4/NF-κB pathway. Meanwhile, the infection induced increased release of RAGE ligands such as S100A8/A9 and HMGB1. These ligands bound to RAGE and formed a positive feedback loop, which further boosted RAGE expression and amplified inflammation. Upon *Brucella* invasion, M1 pro-inflammatory macrophage polarization is preferentially induced, prompting activated macrophages to actively secrete large amounts of HMGB1 [24]. As the most potent endogenous ligand for RAGE, HMGB1 binding continuously activates the NF-κB and MAPK pathways. This activation positively regulates RAGE gene transcription in monocytes and endothelial cells, leading to the upregulation of membrane-bound RAGE expression. Subsequently, membrane-bound RAGE is cleaved by proteases and shed into the bloodstream as soluble RAGE (sRAGE), directly elevating peripheral blood RAGE levels. Furthermore, *Brucella* LPS can form immune complexes with human antibodies such as IgM and IgG. These complexes continuously stimulate dendritic cells and macrophages to secrete pro-inflammatory cytokines, including TNF-α and IL-1β. In turn, TNF-α and IL-1β directly promote RAGE transcription in endothelial cells and macrophages, establishing a cascade amplification loop involving antigen–antibody complexes, inflammatory cytokines, and RAGE. Additionally, in patients with high antibody titers, concurrent Th1/Th2 immune activation often occurs, facilitating massive IFN-γ secretion, which can also significantly upregulate RAGE expression in macrophages. Consequently, serum RAGE levels in acute-phase brucellosis patients are higher than those in the control group, with this difference being more pronounced in the high-titer group.

AGEs are stable and irreversible end products formed by non-enzymatic glycation reactions between reducing sugars (e.g., glucose, fructose) and free amino groups of macromolecules such as proteins, lipids, or nucleic acids in the absence of enzymatic catalysis. The binding of AGEs to specific cell surface-associated receptors can accelerate the oxidation process [31]. AGEs promote the formation of reactive oxygen species (ROS) by regulating the activation pathway of nicotinamide adenine dinucleotide phosphate (NADPH) oxidase and induce the NF-κB pathway, while excessive ROS can further promote the production of endogenous AGEs [32]. In addition, excessive ROS can significantly deplete glutathione (GSH), leading to reduced activity of glyoxalase I (Gly I), which antagonizes AGE accumulation. Meanwhile, the binding of AGEs to their receptors can directly downregulate the expression level of Gly I, thereby increasing the expression levels of AGE precursors and glyoxal, and further promoting AGE formation [33]. Following the binding of AGE receptors to their ligands, intracellular signaling pathways can be activated through phosphatidylinositol 3-kinases (PI3Ks), p38 MAPK, and ERK1/2, along with the activation of cell division cycle 42 (CDC42) and the Rac protein family, ultimately triggering the activation of the pro-inflammatory transcription factor NF-κB and the expression of corresponding pro-inflammatory genes [34]. Among these patients with acute brucellosis, AGEs might bind more extensively to these acute-phase proteins, forming unstable complexes, which leads to a decrease in the level of free AGEs detected by conventional assays. In addition, acute brucellosis induces varying degrees of inflammatory responses. Therefore, the detected serum level of AGEs in the case group was lower than that in the control group.

As an endogenous protective factor, the upregulation of esRAGE expression is generally a negative feedback regulatory mechanism in response to persistent inflammatory stimulation. However, the brucellosis cases included in this study were at the acute phase, when patients were in the early stage of the host immune response, and this protective mechanism of esRAGE might not have been fully activated. Therefore, it was speculated that the serum level of esRAGE does not increase in the early stage of brucellosis.

S100A12 can regulate the expression of various inflammatory factors and co-stimulatory molecules in the body, thereby exerting innate immune functions. Meanwhile, it promotes humoral immune responses through antigen presentation by antigen-presenting cells, leading to the production of immune antibodies that eliminate extracellular pathogens. However, *Brucella* is an intracellular parasitic bacterium, and humoral immunity has a relatively limited role in clearing the pathogen from the body [35]. Therefore, it is hypothesized that the serum level of S100A12 does not increase in patients with acute brucellosis.

This study has several limitations. First, we only measured the circulating concentrations of HMGB1, RAGE, esRAGE, S100A12, and AGEs. We did not conduct validation experiments on the signal transduction pathways involving these cytokines, nor did we directly assess the expression of membrane-bound RAGE, the activation of NF-κB/MAPK, the production of downstream inflammatory cytokines, or cellular responses. Although the mechanistic associations discussed above (such as M1 macrophage activation, HMGB1 release, RAGE signaling, and downstream inflammatory pathways) are biologically plausible and supported by previous literature, they were not directly validated in the present study. Therefore, in future research, we plan to conduct validation experiments focusing on the signal transduction pathways of the HMGB1/RAGE pro-inflammatory axis and related cytokines. Furthermore, we will collaborate with clinicians to incorporate relevant clinical indicators, including blood glucose, body mass index (BMI), oxidative stress markers, and immune cell activation markers, to comprehensively explore the clinical utility of these cytokines in the diagnosis, treatment, and prognostic evaluation of brucellosis patients at different disease stages.

In conclusion, serum levels of HMGB1 and RAGE were significantly elevated in patients with acute brucellosis in Qinghai Province, whereas serum AGEs levels were decreased. These alterations may be associated with host immune defense mechanisms against *Brucella* infection, and these cytokines may interact during the inflammatory response induced by brucellosis.

## Figures and Tables

**Table 1:** Demographic characteristics between the case group and the control group.

Variables	Patients	Controls	*p*
Age (years, $\bar{x}$ ± s)	45.86 ± 10.485	43.63 ± 10.802	0.114
Gender (%)			0.477
Males	71 (64.0)	70 (58.8)	
Females	41 (36.0)	49 (41.2)	
Ethnic (%)			0.187
Han	43 (39.6)	64 (53.9)	
Hui	47 (41.2)	43 (36.1)	
Tu	7 (6.1)	6 (5.0)	
Tibetan	11 (9.6)	4 (3.4)	
Mongolian	1 (0.9)	1 (0.8)	
Salar	2 (0.7)	1 (0.8)	
Dongxiang	1 (0.9)	0 (0.0)	
Occupational (%)			0.019 *
Farmer	105 (93.9)	93 (78.1)	
Herdsman	2 (1.7)	2 (1.7)	
Worker	1 (0.9)	6 (5.0)	
Occupational Population	3 (2.6)	9 (7.6)	
Individual Operator	1 (0.9)	1 (0.8)	
Clerk	0 (0.0)	4 (3.4)	
Others	0 (0.0)	4 (3.4)	

Note: * *p* < 0.05.

**Table 2:** Clinical manifestations of brucellosis patients.

Clinical Manifestations	Number of Cases	Percentage (%)
Fever	64	57.1
Chills	23	20.5
Hyperhidrosis	55	49.1
Fatigue	53	47.3
Arthralgia	63	56.3
Lumbago	41	36.6
Myalgia	14	12.5
Neuralgia	8	7.1
Orchialgia and Swelling	6	5.4

Note: Some brucellosis patients may have multiple clinical manifestations, so the total number of recorded clinical manifestations is greater than the total number of patients.

**Table 3:** RBPT and SAT results between the case group and the control group.

Groups	RBPT		Total	SAT				Total
	Positive	Negative		1/100	1/200	1/400	Negative	
Patients	112	0	112	32	30	50	0	112
Controls	0	119	119	0	0	0	119	119
Total	112	119	231	32	30	50	119	231

**Table 4:** Comparison of serum levels of HMGB1, RAGE, esRAGE, S100A12, and AGEs between the case group and the control group.

	Groups	Males	Females	Total
HMGB1 (ng/mL)	Patients	9.267 ± 3.454	9.052 ± 2.895	9.188 ± 3.249
	Controls	7.527 ± 2.268	7.916 ± 2.549	7.687 ± 2.385
	*t*	3.530	1.980	4.019
	*p*	<0.001 *	0.051	<0.001 *
	*d*	0.595	0.419	0.529
RAGE (ng/mL)	Patients	3.736 ± 0.983	4.040 ± 0.837	3.847 ± 0.940
	Controls	3.413 ± 0.901	3.576 ± 0.970	3.480 ± 0.930
	*t*	2.036	2.403	2.986
	*p*	0.044 *	0.018 *	0.003 *
	*d*	0.343	0.509	0.393
esRAGE (ng/mL)	Patients	8.086 ± 1.781	8.082 ± 1.935	8.085 ± 1.830
	Controls	8.197 ± 2.014	8.325 ± 1.656	8.249 ± 1.869
	*t*	−0.347	−0.640	−0.677
	*p*	0.729	0.524	0.499
	*d*	−0.058	−0.135	−0.089
S100A12 (pg/mL)	Patients	4309 ± 946	3608 ± 1698	4052 ± 1311
	Controls	4146 ± 987	4298 ± 823	4209 ± 923
	*t*	1.004	−2.517	−1.052
	*p*	0.317	0.014 *	0.294
	*d*	0.169	−0.533	−0.139
AGEs (μg/mL)	Patients	313.5 ± 145.8	346.4 ± 140.7	325.5 ± 144.2
	Controls	350.9 ± 85.6	364.6 ± 83.1	356.6 ± 84.5
	*t*	−1.853	−0.765	−2.009
	*p*	0.066	0.446	0.046 *
	*d*	−0.312	−0.162	−0.264

Note: * *p* < 0.05.

**Table 5:** Comparison of serum levels of HMGB1, RAGE, esRAGE, S100A12, and AGEs between the case group with different antibody titers and the control group.

	Groups	*n*	$\bar{x}$ ± s	*F*	*p*	*η* ^2^
HMGB1 (ng/mL)	High Titer Group	50	9.909 ± 3.720	7.978	<0.001 *	0.095
	Medium Titer Group	30	9.046 ± 2.322			
	Low Titer Group	32	8.195 ± 2.999			
	Controls	119	7.687 ± 2.385			
RAGE (ng/mL)	High Titer Group	50	4.003 ± 0.966	3.957	0.009 *	0.050
	Medium Titer Group	30	3.800 ± 0.781			
	Low Titer Group	32	3.649 ± 1.017			
	Controls	119	3.480 ± 0.930			
esRAGE (ng/mL)	High Titer Group	50	8.358 ± 1.988	0.976	0.405	0.013
	Medium Titer Group	30	8.035 ± 1.562			
	Low Titer Group	32	7.704 ± 1.787			
	Controls	119	8.249 ± 1.869			
S100A12 (pg/mL)	High Titer Group	50	4305 ± 1258	2.027	0.111	0.026
	Medium Titer Group	30	3939 ± 1247			
	Low Titer Group	32	3764 ± 1412			
	Controls	119	4209 ± 923			
AGEs (μg/mL)	High Titer Group	50	332.5 ± 158.0	1.533	0.207	0.020
	Medium Titer Group	30	311.9 ± 137.3			
	Low Titer Group	32	327.6 ± 130.8			
	Controls	119	356.6 ± 84.5			

Note: * *p* < 0.05.

**Table 6:** Correlation analysis of serum cytokines HMGB1, RAGE, esRAGE, S100A12, and AGEs in brucellosis patients.

	Groups	HMGB1	RAGE	esRAGE	S100A12	AGEs
HMGB1	Pearson Correlation	1	0.447	0.288	0.156	−0.216
	*p*		<0.001 *	0.005 *	0.126	0.044 *
RAGE	Pearson Correlation	0.447	1	0.602	0.030	0.358
	*p*	<0.001 *		<0.001 *	0.755	<0.001 *
esRAGE	Pearson Correlation	0.288	0.602	1	0.176	0.587
	*p*	0.005 *	<0.001 *		0.090	<0.001 *
S100A12	Pearson Correlation	0.156	0.030	0.176	1	0.098
	*p*	0.126	0.755	0.090		0.340
AGEs	Pearson Correlation	−0.216	0.358	0.587	0.098	1
	*p*	0.044 *	<0.001 *	<0.001 *	0.340	

Note: * *p* < 0.05.

**Table 7:** Correlation analysis of serum cytokines HMGB1, RAGE, esRAGE, S100A12, and AGEs in brucellosis patients with high antibody titers.

	Groups	HMGB1	RAGE	esRAGE	S100A12	AGEs
HMGB1	Pearson Correlation	1	0.285	0.085	0.033	−0.363
	*p*		0.090	0.703	0.822	0.032 *
RAGE	Pearson Correlation	0.285	1	0.512	−0.044	0.339
	*p*	0.090		<0.001 *	0.762	0.032 *
esRAGE	Pearson Correlation	0.085	0.512	1	0.100	0.556
	*p*	0.703	<0.001 *		0.610	<0.001 *
S100A12	Pearson Correlation	0.033	−0.044	0.100	1	0.079
	*p*	0.822	0.762	0.610		0.703
AGEs	Pearson Correlation	−0.363	0.339	0.556	0.079	1
	*p*	0.032 *	0.032 *	<0.001 *	0.703	

Note: * *p* < 0.05.

**Table 8:** Correlation analysis of serum cytokines HMGB1, RAGE, esRAGE, S100A12, and AGEs in brucellosis patients with medium antibody titers.

	Groups	HMGB1	RAGE	esRAGE	S100A12	AGEs
HMGB1	Pearson Correlation	1	0.495	0.408	−0.081	−0.131
	*p*		0.016 *	0.042 *	0.766	0.610
RAGE	Pearson Correlation	0.495	1	0.540	−0.053	0.391
	*p*	0.016 *		0.010 *	0.779	0.042 *
esRAGE	Pearson Correlation	0.408	0.540	1	−0.057	0.616
	*p*	0.042 *	0.010 *		0.766	<0.001 *
S100A12	Pearson Correlation	−0.081	−0.053	−0.057	1	0.097
	*p*	0.766	0.779	0.766		0.766
AGEs	Pearson Correlation	−0.131	0.391	0.616	0.097	1
	*p*	0.610	0.042 *	<0.001 *	0.766	

Note: * *p* < 0.05.

**Table 9:** Correlation analysis of serum cytokines HMGB1, RAGE, esRAGE, S100A12, and AGEs in brucellosis patients with low antibody titers.

	Groups	HMGB1	RAGE	esRAGE	S100A12	AGEs
HMGB1	Pearson Correlation	1	0.666	0.566	0.412	0.015
	*p*		<0.001 *	0.003 *	0.048 *	0.936
RAGE	Pearson Correlation	0.666	1	0.763	0.095	0.380
	*p*	<0.001 *		<0.001 *	0.604	0.052
esRAGE	Pearson Correlation	0.566	0.763	1	0.392	0.648
	*p*	0.003 *	<0.001*		0.052	<0.001 *
S100A12	Pearson Correlation	0.412	0.095	0.392	1	0.119
	*p*	0.048 *	0.604	0.052		0.604
AGEs	Pearson Correlation	0.015	0.380	0.648	0.119	1
	*p*	0.936	0.052	<0.001 *	0.604	

Note: * *p* < 0.05.

## Data Availability

All the relevant data have been provided within the manuscript.
